# Health-related quality of life in benign thyroid diseases assessed by ThyPRO

**DOI:** 10.1530/EC-26-0097

**Published:** 2026-10-27

**Authors:** Nadia Sawicka-Gutaj, Aleksandra Derwich-Rudowicz, Barbara Bromińska, Joanna Maciejewska, Kacper Nijakowski, Paulina Kozłowska, Oliwier Adamczak, Zachariasz Rytelewski, Angelos Kyriacou, Marek Ruchała

**Affiliations:** ^1^ Department of Endocrinology, Metabolism and Internal Medicine, Poznan University of Medical Sciences, Poznań, Poland; ^2^ Department of Conservative Dentistry, Poznan University of Medical Sciences, Poznań, Poland; ^3^ School of Medicine, European University Cyprus, Nicosia, Cyprus; ^4^ Diabetes, Endocrinology and Obesity Medicine, Salford Royal Hospital, Northern Care Alliance NHS Foundation Trust, Salford, Greater Manchester, UK

**Keywords:** quality of life, patient reported outcomes, Graves’ disease, hyperthyroidism, hypothyroidism, goiter

## Abstract

**Purpose:**

Benign thyroid diseases are common conditions that often require long-term treatment. In some patients, symptoms persist despite normalization of biochemical parameters, significantly affecting quality of life (QoL). This study evaluated treatment-associated changes in QoL using the thyroid patient reported outcome (ThyPRO) questionnaire and summarized differences in QoL profiles reported across thyroid conditions.

**Methods:**

A systematic review and meta-analysis was performed according to PRISMA guidelines. PubMed, Scopus, and Embase were searched up to April 2024. Data were extracted and synthesized using standardized mean differences.

**Results:**

Twenty-five studies were included. Significant pre-post-treatment improvements in overall QoL and selected symptom domains were observed. In Graves’ disease, improvements were observed in hyperthyroid symptoms, anxiety, impaired daily life, and overall QoL. In non-toxic goiter, improvements were observed in goiter symptoms, cosmetic complaints, anxiety, tiredness, and overall QoL. In toxic nodular goiter, significant improvements were observed in hyperthyroid and goiter symptoms and overall QoL, whereas several other domains did not change significantly. In hypothyroidism, significant improvements were observed in selected domains, including tiredness, impaired daily life, mood-related symptoms, and overall QoL. Analyses comparing ThyPRO scores between patient groups showed differences in selected domains; these comparisons were interpreted descriptively.

**Conclusion:**

Treatment was associated with improvements in QoL across the analyzed benign thyroid conditions, varying by disease group. Given the heterogeneity of the included studies and the absence of head-to-head comparisons of treatment efficacy across conditions, these findings should be interpreted descriptively. Disease-specific QoL assessment may help clinicians identify persistent symptom domains requiring attention during follow-up.

## Introduction

Benign thyroid diseases are among the most commonly diagnosed endocrine conditions. They are highly prevalent in the general population, particularly among women, and their incidence increases with age (1, 2). These disorders comprise a heterogeneous group of non-malignant thyroid conditions that may be broadly classified into autoimmune diseases, such as Graves’ disease or Hashimoto’s thyroiditis, thyroid dysfunctions manifesting as non-autoimmune hypothyroidism or hyperthyroidism, and structural thyroid abnormalities, particularly non-toxic nodular goiters.

These diseases are usually not life threatening, but they are associated with a broad spectrum of somatic and psychological symptoms that significantly affect patients’ well-being. Commonly reported complaints include chronic fatigue, changes in physical appearance such as unintentional weight changes or deterioration in skin and hair condition, as well as various psychological symptoms ranging from agitation and emotional lability in hyperthyroidism to depression in hypothyroidism. Cognitive impairment is also frequently reported (3, 4, 5, 6). The consequences of these diseases extend beyond the physical and emotional domains, affecting social functioning as well. Chronic fatigue, depression, and changes in appearance may lead to limitations in professional work and sexual activity (7, 8).

Due to the chronic nature of benign thyroid diseases, many patients require lifelong treatment and, unfortunately, some symptoms may persist even after euthyroidism is achieved. For these reasons, the assessment of treatment effectiveness has started to include not only biochemical parameters such as thyroid-stimulating hormone (TSH) levels but also patients’ quality of life (QoL).

For this purpose, standardized tools for assessing QoL have been developed, with thyroid patient reported outcome (ThyPRO) designed for benign thyroid diseases. The questionnaire serves as the recommended primary instrument for QoL assessment in this patient group because of its high validity, reliability, and sensitivity (9). It contains 85 items grouped into 13 scales covering somatic and psychological symptoms, as well as aspects related to social functioning and general well-being. This tool includes domains specific to benign thyroid diseases typically absent from generic questionnaires, such as goiter symptoms, hyper- and hypothyroid symptoms, eye problems, and cosmetic concerns. Responses are given on a five-point Likert scale and converted to a 0–100 point scale, with higher scores indicating a greater negative impact of thyroid disease on the patient’s quality of life (9, 10). Numerous studies have used ThyPRO to assess the quality of life in patients with various thyroid disorders. However, most of these studies have focused on individual disorders. As a result, the findings remain fragmented, making it difficult to draw broader conclusions regarding treatment-related changes in quality of life. For this reason, the aim of our study was to perform a comprehensive analysis of the available evidence on changes in QoL measured using the ThyPRO questionnaire following pharmacological and surgical treatment in patients with selected benign thyroid diseases, including Graves’ disease, Graves’ orbitopathy, Hashimoto’s thyroiditis, hypothyroidism, and toxic or non-toxic goiter (11, 12, 13, 14, 15, 16, 17, 18, 19, 20, 21, 22, 23, 24, 25, 26, 27, 28, 29, 30, 31, 32, 33, 34, 35).

## Materials and methods

### Study design and reporting standards

This study was designed as a systematic review and meta-analysis conducted in accordance with the Preferred Reporting Items for Systematic Reviews and Meta-Analyses (PRISMA) guidelines. The methodology was defined *a priori* and followed a structured protocol specifying the research question, eligibility criteria, search strategy, study selection process, data extraction, quality assessment, and statistical analysis. The review focused on studies assessing health-related quality of life in patients with benign thyroid diseases using the ThyPRO questionnaire. All stages of the review process, including literature search, screening, data extraction, and risk-of-bias assessment, were performed in a transparent and reproducible manner to ensure methodological rigor and minimize bias.

### Data sources and search strategy

This systematic review was based on studies published from 2010 to 01 April 2024. Figure 1 presents the study design and methodology. A comprehensive literature search was performed across three databases: PubMed, Scopus, and Embase. The following search algorithm was applied: (‘ThyPRO’ OR ‘thyroid-specific patient-reported outcome’ OR ‘quality of life’) AND (‘benign thyroid disease’ OR ‘hyperthyroidism’ OR ‘Graves’ OR ‘nodular thyroid goiter’ OR ‘hypothyroidism’ OR ‘Hashimoto’ OR ‘autoimmune thyroid disease’). The literature search was independently performed by three investigators (ADR, BB, and JM). After the removal of duplicates, titles and abstracts were screened for eligibility. Full-text articles were assessed against prespecified inclusion and exclusion criteria. Discrepancies during the screening process were resolved by consulting the clinical experts and methodologists. The specific criteria for inclusion and exclusion of relevant studies in terms of PI(E)COS (‘population’, ‘intervention’/‘exposure’, ‘comparison’, ‘outcomes’, and ‘study design’) are provided in Table 1. A detailed search flowchart is shown in Fig. 1.

**Figure 1:**
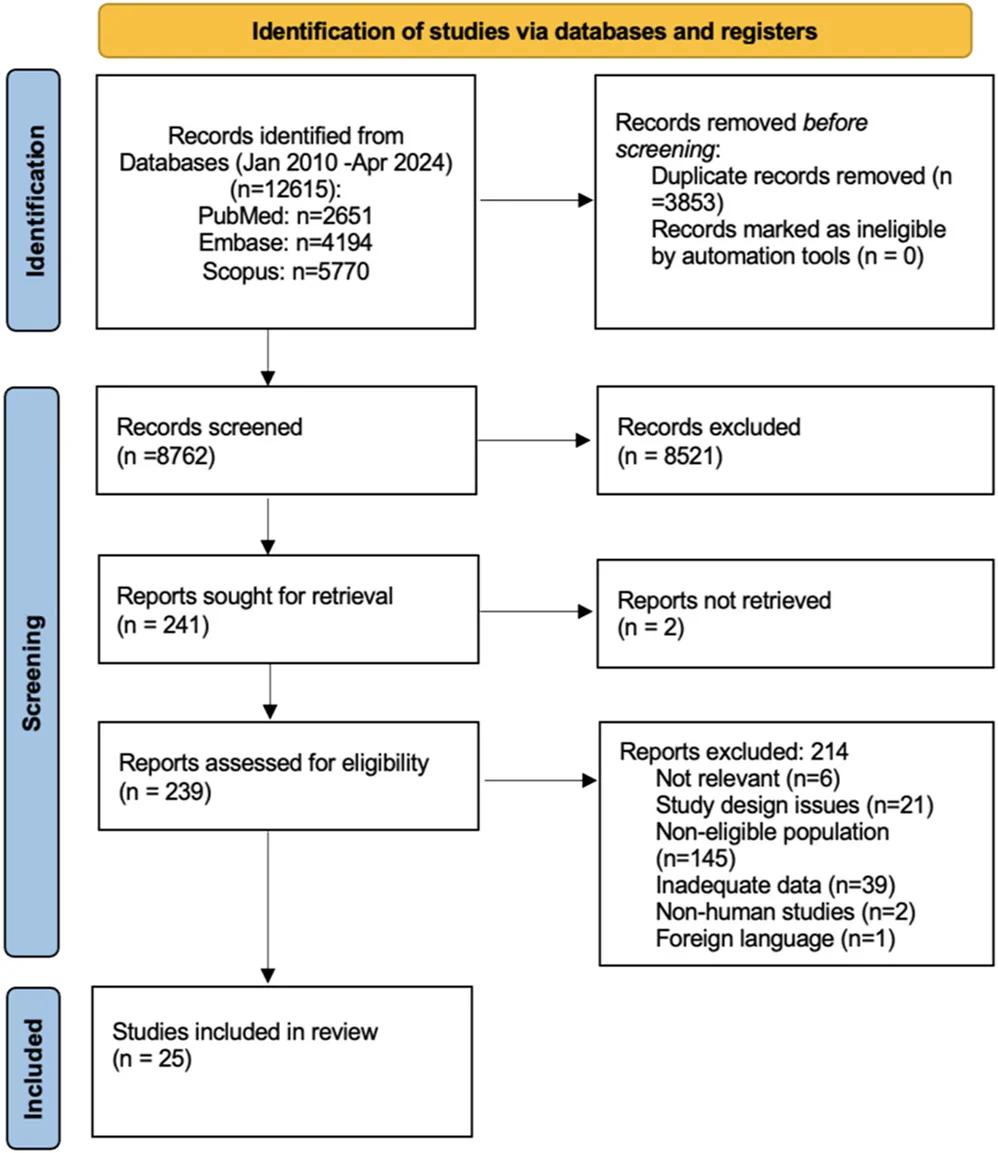
PRISMA flow diagram presenting search strategy. This systematic review was conducted based on articles published from 2010 up to 01 April 2024. A comprehensive literature search was performed across three databases: PubMed, Scopus, and Embase. After the identification process, duplicate records were removed. Record counts mentioned in the screening step reflect post-deduplication totals. Inclusion and exclusion criteria according to the PICOS are described in Table 1.

**Table 1:** Inclusion and exclusion criteria according to the PICOS.

Parameter	Inclusion criteria	Exclusion criteria
Population	Adult patients (≥18 years) with benign thyroid diseases, including hyperthyroidism, hypothyroidism, Graves’ disease, Hashimoto’s thyroiditis, nodular thyroid disease	Patients aged < 18 years
		Patients with thyroid malignancy
		*In vitro*, *in vivo*, or animal studies
Intervention/exposure	Any clinical management or disease state in benign thyroid disease, including medical treatment (e.g. antithyroid drugs, levothyroxine), radioiodine therapy, surgery, or conservative management, where ThyPRO is assessed	
Comparison	Different thyroid disease groups	
	Different treatment modalities	
	Studies without control groups (single-arm or observational studies)	
Outcomes	Primary outcome:	Studies not using ThyPRO as an outcome measure
	Thyroid-related quality of life measured using ThyPRO (any version) or ThyPRO-39	
	Secondary outcome:	Studies not reporting sufficient data on ThyPRO domains or total scores
	Associations between ThyPRO scores and disease severity, thyroid function tests, treatment modality, or disease duration	
Study design	Case–control, RTC, cohort, and cross-sectional studies	Literature reviews, case reports, expert opinion, letters to the editor, conference reports
	Published from the inception date of each database until April 1, 2024	Not published in English

### Data extraction

Data were extracted using a predefined and piloted data extraction form. The following information was collected from each eligible study: first author, year of publication, country, study design, sample size, demographic characteristics of the study population (age and sex), type of benign thyroid disease, treatment modality, duration of follow-up, laboratory parameters, and comparator group where applicable. Outcome data included ThyPRO domain scores and overall quality-of-life scores at baseline and follow-up, or comparisons between patient groups and controls. When reported, means and standard deviations were extracted directly; otherwise, these were estimated from medians, interquartile ranges, confidence intervals, or standard errors using established statistical methods.

### Quality assessment and critical appraisal for the systematic review of the included studies

The methodological quality of the included studies was evaluated independently by two reviewers using the Study Quality Assessment Tools developed by the National Heart, Lung, and Blood Institute (NHLBI) of the National Institutes of Health (NIH), tailored to the design of each included study. Each study was assessed across key domains, including clarity of objectives, eligibility criteria, sample size justification, outcome measurement, and adequacy of statistical analyses (Supplementary Fig. S1 (see section on Supplementary materials given at the end of the article)).

### Statistical analysis

The meta-analysis was performed for assessed ThyPRO domains within four disease groups: Graves’ disease, non-toxic goiter, hypothyroidism, and toxic nodular goiter. For pre-post-treatment analyses, pooled changes were quantified using standardized mean differences (SMDs) with corresponding 95% confidence intervals (95% CIs) and *P*-values. Negative SMDs indicate improvement after treatment because higher ThyPRO scores reflect worse QoL. For analyses comparing ThyPRO scores between patient groups, SMDs were calculated according to the group contrasts presented in Supplementary Table S1 (Supplementary Table S1 provides the full-pooled estimates for analyses comparing ThyPRO profiles across thyroid conditions); these estimates were interpreted descriptively and not as evidence of comparative treatment efficacy. Pooled pre-post-treatment estimates were calculated using statistical formulas for paired analyses (dependent samples). The number of contributing studies (*k*), number of participants (*n*), and heterogeneity statistic (*I*^2^) are reported with the corresponding pooled estimates in Tables 3, 4, 5, 6, and Supplementary Table S1. For treatment-effect analyses with multiple follow-up measurements, the 6-month follow-up was used to avoid non-independent observations. Given the clinical and methodological heterogeneity of the included studies, random-effects models were applied for all meta-analyses. Egger’s test was used to assess publication bias. A significance level of alpha = 0.05 was adopted for all tests. All analyses were conducted using MedCalc Statistical Software, version 22.014 (MedCalc Software Ltd, Belgium).

## Results

### Overview of included studies

This systematic review included 25 papers examining changes in QoL following treatment in patients with benign thyroid diseases using the ThyPRO questionnaire. The studies included in the analysis, along with patient characteristics and treatment modalities, are summarized in Table 2.

**Table 2:** General characteristics of included studies.

Study	Country	Study design	*n* (F)	Age (years)	Diagnosis	Treatment
Azaria *et al.* (11)	India	PCS	50 (34)	43.30 ± 11.61	Benign goiter	Thyroidectomy
Bukvic *et al.* (12)	Serbia	PLCCS	132 (112)	52.7 ± 12.5	Euthyroid goiter: 104 (78.8%); toxic adenoma/toxic goiter: 27 (20.5%); hypothyroid goiter: 1 (0.8%)	Thyroidectomy
Bukvic *et al.* (13)	Serbia	CS	100 (88)	48.77 ± 13.13	Non-toxic goiter: 51 (51%); toxic goiter: 20 (20%); GD with TED: 19 (19%); GD without TED: 3 (3%); Hashimoto disease: 7 (7%)	LT4: 8 (8%); antithyroid drugs: 30 (30%); none: 62 (62%)
Bukvic *et al.* (14)	Serbia	PLCCS	59 (54)	Toxic nodular goiter: 55.9 ± 12.7; GD: 42.9 ± 13.4	Toxic nodular goiter: 28 (47.5%); GD: 31 (52.5%)	Thyroidectomy
Cramon *et al.* (15)	Denmark	PCS	111 (88)	Mean: 53; Q1–Q3: 46–61	Euthyroid nodular goiter	Hemithyroidectomy: 59 (53%); total thyroidectomy: 13 (12%); radioactive iodine: 35 (32%); ethanol sclerotherapy (thyroid cysts): 4 (4%)
Cramon *et al.* (16)	Denmark	PCS	156 (127)	GD: median 47; range (22-?); toxic nodular goiter: median 62; range (23–84)	GD: 88 (56%); toxic nodular goiter: 68 (44%)	Antithyroid drugs – GD: 78 (89%), toxic nodular goiter: 10 (15%); radioiodine therapy – GD: 2 (2%), toxic nodular goiter: 50 (74%); hemithyroidectomy – GD: 1 (1%), toxic nodular goiter: 3 (4%); total thyroidectomy – GD: 7 (8%), toxic nodular goiter: 5 (7%)
Johri *et al.* (17)	India	LS	93 (76)	Endoscopic: 30.7 ± 9.2; conventional: 33.0 ± 9.6	Euthyroid nodular goiter	Endoscopic hemithyroidectomy: 41 (44%); conventional hemithyroidectomy: 52 (56%)
Martino *et al.* (18)	Italy	CSS	21 (16)	58.1 ± 12.3	Hashimoto’s thyroiditis	LT4
Michaelsson *et al.* (19)	Denmark	OLCS	23 (21)	47 (33–76)	Hypothyroidism – autoimmunity: 17 (73.9%); radioiodine therapy: 4 (17.4%); thyroidectomy: 1 (4.3%); congenital: 1 (4.3%)	LT4/LT3
Mishra *et al.* (20)	India	PCS	100 (81)	40.5 ± 13.2	Multinodular goiter: 82 (82%); diffuse goiter: 18 (18%)	Hemithyroidectomy: 46 (46%); thyroidectomy: 54 (54%)
Molewijk *et al.* (21)	The Netherlands	OS*	1,667 (1,528)	50.1 ± 12.7	Hypothyroidism – Hashimoto’s disease: 619 (37.1%); RAI for GD: 148 (8.9%); resection thyroid gland (malignant): 56 (3.4%); resection thyroid gland (non-malignant): 60 (3.6%); congenital hypothyroidism: 34 (2.0%); pituitary tumor/dysfunction: 19 (1.1%); unknown: 521 (31.3%); other: 210 (12.6%)	LT4 only: 1,221 (80.5%); LT4 + LT3: 118 (7.8%); LT4 + DTE: 79 (5.2%); DTE: 91 (6.0%), LT4 + LT3 + DTE: 3 (0.2%); LT3 only: 2 (0.1%); LT3 + DTE: 2 (0.1%)
Oddo *et al.* (22)	Italy	PIS	14 (12)	55 ± 10	Euthyroid nodular goiter	Laser ablation
Recker *et al.* (23)	Germany	PIS	28 (no data)	66 (60–75); 26 (19–35)	Subclinical hypothyroidism >60 years old and <40 years old	LT4
Rigó *et al.* (24)	Hungary	CS	291 (280)	45.1 ± 12.0	Hashimoto’s thyroiditis: 240 (82.5%), GD: 51 (17.5%)	n/a
Sorensen *et al.* (25)	Denmark	PCS	106 (91)	51 ± 13	Non-toxic multinodular goiter	Total thyroidectomy: 23 (22%); hemithyroidectomy: 77 (73%); isthmectomy: 6 (6%)
Sorensen *et al.* (26)	Denmark	PCS	65 (48)	55 ± 13	Multinodular goiter – cervical: 51 (78%); intrathoracic: 14 (22%)	Thyroidectomy
Sorensen *et al.* (27)	Denmark	PCS	54 (48)	50 ± 13	Recurrent cystic thyroid nodule: 37 (69%); solid nodule: 3 (6%); mixed character: 14 (25%)	Laser thermal ablation
Taïeb *et al.* (28)	France	OLRCT	94 (71)	Preventive LT4: 48.8 ± 13.5; observation without LT4: 47.1 ± 12.9	Graves’ hyperthyroidism undergoing radioiodine therapy	Preventive LT4: 46 (49%); observation without LT4: 48 (51%)
Törring *et al.* (29)	Sweden	CSS	975 (794)	43 (33–55); 54 (45–63); 35 (29–46)	GD	Antithyroid drugs only: 347 (36%); RAI: 395 (41); thyroidectomy: 233 (24)
Tuzil *et al.* (30)	Czech Republic	OS	253 (253)	31 ± 5	Subclinical hypothyroidism: 61 (26%); euthyroid: 174 (74%)	LT4
Watt *et al.* (31)	Denmark	PCS	435 (361)	54 (42–63)	Hyperthyroid rendered euthyroid: 66 (16%); hypothyroid rendered euthyroid: 84 (19%); clinically noticeable goiter treated with surgery or radioactive iodine: 62 (14%); other diagnosis: 223 (51%)	L-T4: 111 (26); antithyroid medication: 86 (20); aspiration of thyroid cyst: 4 (1); glucocorticoid pulse therapy: 2 (0); other immunosuppressive treatment: 4 (1); hemithyroidectomy: 64 (15); total thyroidectomy: 37 (9); RAI: 127 (29)
Watt *et al.* (32)	Denmark	CSS	838 (728)	n/a	Non-toxic goiter: 258 (31%); toxic nodular goiter: 142 (17%); Graves’ hyperthyroidism: 158 (19%), Graves’ orbitopathy: 91 (11%); autoimmune hypothyroidism: 189 (23%)	n/a
Winther *et al.* (33)	Denmark	PCS	78 (70)	47 (18–91)	Hypothyroidism, autoimmune thyroiditis	LT4
Zahan *et al.* (34)	Romania	PCS	130 (115)	53 ± 11	Non-toxic goiter: 41 (32%); toxic nodular goiter: 9 (7%); GD with/without TED: 19 (15%); Hashimoto’s thyroiditis: 46 (35%); other thyroid diagnoses: 15 (12%)	No treatment: 64 (49%); LT4: 40 (31%); antithyroid drugs: 23 (18%); others: 3 (2%)
Zivaljevic *et al.* (35)	Serbia	PCS	143 (99)	52.6 ± 12.9; 52.2 ± 10.9	Nodular goiter: 116 (81%); Hashimoto thyroiditis: 27 (19%)	Nodular goiter – thyroidectomy: 73, hemithyroidectomy: 43; Hashimoto thyroiditis – thyroidectomy: 24, hemithyroidectomy: 3

DTE, dessiccated thyroid extract; F, females; IQR, interquartile range; LT3, liothyronine; LT4, levothyroxine; *n*, total number of participants; n/a, not applicable (data not available); GD, Graves’ disease; RAI, radioactive iodine; SD, standard deviation; TED, thyroid eye disease; PCS, prospective cohort study; PLCCS, prospective longitudinal case–control study; CS, cohort study; CSS, cross-sectional study; OLRCT, open label randomized controlled study; OS, observational study; LS, longitudinal study; OLCS, open-label cohort study; PIS, Prospective interventional study.

* Using an online survey.

### Pooled analysis of ThyPRO domains across various thyroid diseases following treatment

Pooled pre-post-treatment estimates for ThyPRO domains within each thyroid disease group are presented in Tables 3, 4, 5, 6.

**Table 3:** Pooled standardized mean differences for ThyPRO domains in patients with Graves’ disease following treatment (negative SMD indicates improvement after treatment).

ThyPRO domain	SMD	SE	95% CI	*P*-value	*I* ^2^	*k*	*n*	Egger’s test	Reference
Goiter symptoms	−0.778	0.247	−1.262; −0.293	0.002*	86.42	4	279	0.014^†^	14, 16, 28, 31
Hyperthyroid symptoms	−1.092	0.090	−1.269; −0.914	<0.001*	0.00	4	279	0.947	14, 16, 28, 31
Hypothyroid symptoms	−0.335	0.091	−0.514; −0.155	<0.001*	12.07	4	279	0.561	14, 16, 28, 31
Eye symptoms	−0.314	0.144	−0.596; −0.032	0.029*	62.47	4	279	0.170	14, 16, 28, 31
Tiredness	−0.632	0.453	−1.521; 0.257	0.163	95.98	4	279	0.433	14, 16, 28, 31
Cognitive complaints	−0.391	0.085	−0.558; −0.224	<0.001*	0.00	4	279	0.181	14, 16, 28, 31
Anxiety	−0.779	0.100	−0.975; −0.584	<0.001*	20.27	4	279	0.246	14, 16, 28, 31
Depressive symptoms	−0.498	0.086	−0.666; −0.330	<0.001*	0.00	4	279	0.246	13, 15, 27, 30
Emotional susceptibility	−0.755	0.087	−0.926; −0.583	<0.001*	0.00	4	279	0.198	14, 16, 28, 31
Impaired social life	−0.519	0.147	−0.808; −0.231	<0.001*	63.60	4	279	0.611	14, 16, 28, 31
Impaired daily life	−0.732	0.087	−0.903; −0.561	<0.001*	0.00	4	279	0.361	14, 16, 28, 31
Impaired sex life	−0.447	0.103	−0.649; −0.245	<0.001*	28.04	4	279	0.151	14, 16, 28, 31
Cosmetic complaints	−0.346	0.170	−0.680; −0.012	0.042*	73.02	4	279	0.114	14, 16, 28, 31
Overall QoL	−0.914	0.109	−1.128; −0.701	<0.001*	0.00	3	185	0.756	14, 16, 31

SMD, standardized mean difference; SE, standard error; CI, confidence interval; *k*, number of contributing studies; *n*, total number of participants; QoL, quality of life.

* Significant pooled standardized mean differences for *P* < 0.05.

^†^ Significant publication bias for *P* < 0.05.

**Table 4:** Pooled standardized mean differences for ThyPRO domains in patients with non-toxic goiter following treatment (negative SMD indicates improvement after treatment).

ThyPRO domain	SMD	SE	95% CI	*P*-value	*I* ^2^	*k*	*n*	Egger’s test	Reference
Goiter symptoms	−1.478	0.122	−1.716; −1.239	<0.001*	75.01	10	732	0.761	11, 12, 15, 20, 22, 25, 26, 27, 31, 35
Hyperthyroid symptoms	−0.590	0.128	−0.842; −0.338	<0.001*	70.21	6	461	0.559	11, 12, 15, 22, 31, 35
Hypothyroid symptoms	−0.195	0.061	−0.315; −0.075	0.001*	4.78	8	567	0.163	11, 12, 15, 22, 25, 27, 31, 35
Eye symptoms	−0.342	0.086	−0.511; −0.173	<0.001*	28.84	6	407	0.809	11, 12, 15, 22, 31, 35
Tiredness	−0.779	0.183	−1.138; −0.421	<0.001*	91.75	11	825	0.236	11, 12, 15, 17, 20, 22, 25, 26, 27, 31, 35
Cognitive complaints	−0.297	0.119	−0.530; −0.064	0.012*	79.93	10	760	0.914	11, 12, 15, 17, 20, 22, 25, 27, 31, 35
Anxiety	−0.667	0.097	−0.857; −0.477	<0.001*	71.03	11	825	0.235	11, 12, 15, 17, 20, 22, 25, 26, 27, 31, 35
Depressive symptoms	−0.586	0.202	−0.983; −0.190	0.004*	92.81	10	760	0.592	11, 12, 15, 17, 20, 22, 25, 27, 31, 35
Emotional susceptibility	−0.564	0.216	−0.989; −0.139	0.009*	93.70	10	760	0.904	11, 12, 15, 17, 20, 22, 25, 27, 31, 35
Impaired social life	−0.564	0.178	−0.913; −0.215	0.002*	90.78	10	760	0.249	11, 12, 15, 17, 20, 22, 25, 27, 31, 35
Impaired daily life	−0.399	0.095	−0.586; −0.211	<0.001*	71.26	11	825	0.846	11, 12, 15, 17, 20, 22, 25, 26, 27, 31, 35
Impaired sex life	−0.341	0.144	−0.624; −0.057	0.018*	83.10	8	617	0.863	11, 12, 15, 22, 25, 27, 31, 35
Cosmetic complaints	−0.667	0.151	−0.962; −0.372	<0.001*	86.25	9	719	0.754	11, 12, 15, 20, 22, 25, 27, 31, 35
Overall QoL	−0.854	0.175	−1.198; −0.510	<0.001*	85.39	7	527	0.808	11, 12, 15, 22, 25, 31, 35

SMD, standardized mean difference; SE, standard error; CI, confidence interval; *k*, number of contributing studies; *n*, total number of participants; QoL, quality of life.

* Significant pooled standardized mean differences for *P* < 0.05.

**Table 5:** Pooled standardized mean differences for ThyPRO domains in patients with hypothyroidism following treatment (negative SMD indicates improvement after treatment).

ThyPRO domain	SMD	SE	95% CI	*P*-value	*I* ^2^	*k*	*n*	Egger’s test	Reference
Goiter symptoms	−0.452	0.308	−1.057; 0.153	0.143	91.57	4	283	0.368	23, 31, 33, 35
Hyperthyroid symptoms	−0.229	0.177	−0.577; 0.120	0.197	65.17	3	205	0.545	23, 31, 35
Hypothyroid symptoms	−0.410	0.174	−0.752; −0.069	0.019*	79.22	5	346	0.966	19, 23, 31, 33, 35
Eye symptoms	−0.210	0.084	−0.374; −0.045	0.013*	0.00	4	283	0.117	23, 31, 33, 35
Tiredness	−0.542	0.118	−0.774; −0.311	<0.001*	54.23	5	346	0.366	19, 23, 31, 33, 35
Cognitive complaints	−0.229	0.076	−0.378; −0.080	0.003*	0.00	5	346	0.361	19, 23, 31, 33, 35
Anxiety	−0.410	0.077	−0.560; −0.260	<0.001*	0.00	5	346	0.602	19, 23, 31, 33, 35
Depressive symptoms	−0.258	0.101	−0.455; −0.060	0.011*	39.50	5	346	0.800	19, 23, 31, 33, 35
Emotional susceptibility	−0.402	0.089	−0.576; −0.227	<0.001*	23.31	5	346	0.416	19, 23, 31, 33, 35
Impaired social life	−0.175	0.128	−0.427; 0.077	0.174	62.41	5	346	0.119	19, 23, 31, 33, 35
Impaired daily life	−0.513	0.077	−0.664; −0.362	<0.001*	0.00	5	346	0.038	19, 23, 31, 33, 35
Impaired sex life	−0.290	0.085	−0.457; −0.122	0.001*	2.29	4	283	0.312	23, 31, 33, 35
Cosmetic complaints	−0.311	0.089	−0.486; −0.136	0.001*	8.45	4	283	0.207	23, 31, 33, 35
Overall QoL	−0.729	0.195	−1.111; −0.346	<0.001*	76.00	4	252	0.093	19, 31, 33, 35

SMD, standardized mean difference; SE, standard error; CI, confidence interval; *k*, number of contributing studies; *n*, total number of participants; QoL, quality of life.

* Significant pooled standardized mean differences for *P* < 0.05.

**Table 6:** Pooled standardized mean differences for ThyPRO domains in patients with toxic nodular goiter following treatment (negative SMD indicates improvement after treatment).

ThyPRO domain	SMD	SE	95% CI	*P*-value	*I* ^2^	*k*	*n*	Egger’s test	Reference
Goiter symptoms	−1.590	0.635	−2.840; −0.340	0.013*	94.50	3	135	0.113	12, 14, 16
Hyperthyroid symptoms	−1.047	0.412	−1.859; −0.236	0.012*	89.02	3	135	0.229	12, 14, 16
Hypothyroid symptoms	−0.243	0.121	−0.481; −0.004	0.046*	0.00	3	135	0.136	12, 14, 16
Eye symptoms	−0.418	0.122	−0.659; −0.178	0.001*	0.00	3	135	0.225	12, 14, 16
Tiredness	−0.818	0.320	−1.447; −0.188	0.011*	82.82	3	135	0.093	12, 14, 16
Cognitive complaints	−0.119	0.121	−0.357; 0.119	0.325	0.00	3	135	0.068	12, 14, 16
Anxiety	−0.564	0.123	−0.807; −0.322	<0.001*	0.00	3	135	0.135	12, 14, 16
Depressive symptoms	−0.364	0.122	−0.604; −0.124	0.003*	0.00	3	135	0.289	12, 14, 16
Emotional susceptibility	−0.529	0.360	−1.238; 0.181	0.143	86.93	3	135	0.534	12, 14, 16
Impaired social life	−0.495	0.259	−1.005; 0.016	0.057	75.36	3	135	0.502	12, 14, 16
Impaired daily life	−0.321	0.157	−0.631; −0.012	0.042*	36.20	3	135	0.528	12, 14, 16
Impaired sex life	−0.289	0.121	−0.528; −0.050	0.018*	0.00	3	135	0.739	12, 14, 16
Cosmetic complaints	−0.764	0.268	−1.292; −0.236	0.005*	75.92	3	135	0.138	12, 14, 16
Overall QoL	−1.120	0.317	−1.745; −0.496	<0.001*	81.30	3	135	0.145	12, 14, 16

SMD, standardized mean difference; SE, standard error; CI, confidence interval; *k*, number of contributing studies; *n*, total number of participants; QoL, quality of life.

* Significant pooled standardized mean differences for *P* < 0.05.

In Graves’ disease, significant pre-post-treatment improvements were observed in hyperthyroid symptoms (SMD = −1.092; 95% CI: −1.269 to −0.914; *P* < 0.001), overall QoL (SMD = −0.914; 95% CI: −1.128 to −0.701; *P* < 0.001), impaired daily life (SMD = −0.732; 95% CI: −0.903 to −0.561; *P* < 0.001), and anxiety (SMD = −0.779; 95% CI: −0.975 to −0.584; *P* < 0.001). Significant changes were also observed in emotional susceptibility, depressive symptoms, and goiter symptoms, whereas tiredness did not change significantly (*P* = 0.163).

In patients with non-toxic goiter, significant pre-post-treatment improvements were observed across all assessed domains. Goiter symptoms improved with an SMD of −1.478 (95% CI: −1.716 to −1.239; *P* < 0.001), overall QoL with an SMD of −0.854 (95% CI: −1.198 to −0.510; *P* < 0.001), cosmetic complaints with an SMD of −0.667 (95% CI: −0.962 to −0.372; *P* < 0.001), anxiety with an SMD of −0.667 (95% CI: −0.857 to −0.477; *P* < 0.001), and tiredness with an SMD of −0.779 (95% CI: −1.138 to −0.421; *P* < 0.001). Significant changes were also observed in the remaining assessed domains (Table 4).

In hypothyroidism, significant pre-post-treatment improvements were observed in tiredness (SMD = −0.542; 95% CI: −0.774 to −0.311; *P* < 0.001), overall QoL (SMD = −0.729; 95% CI: −1.111 to −0.346; *P* < 0.001), and impaired daily life (SMD = −0.513; 95% CI: −0.664 to −0.362; *P* < 0.001). Significant changes were also observed in emotional susceptibility, anxiety, depressive symptoms, cognitive complaints, hypothyroid symptoms, eye symptoms, impaired sex life, and cosmetic complaints. Changes in goiter symptoms, hyperthyroid symptoms, and impaired social life were not statistically significant (Table 5).

In toxic nodular goiter, significant pre-post-treatment improvements were observed in goiter symptoms (SMD = −1.590; 95% CI: −2.840 to −0.340; *P* = 0.013), hyperthyroid symptoms (SMD = −1.047; 95% CI: −1.859 to −0.236; *P* = 0.012), overall QoL (SMD = −1.120; 95% CI: −1.745 to −0.496; *P* < 0.001), tiredness (SMD = −0.818; 95% CI: −1.447 to −0.188; *P* = 0.011), anxiety, depressive symptoms, impaired daily life, impaired sex life, cosmetic complaints, hypothyroid symptoms, and eye symptoms. Changes in cognitive complaints, emotional susceptibility, and impaired social life were not statistically significant (Table 6).

### Comparative analysis of ThyPRO domains across thyroid conditions

The comparative analysis of ThyPRO domains across thyroid conditions revealed significant differences in symptom burden and QoL between patients and healthy controls, as well as among the disease groups themselves. Full-pooled estimates, 95% confidence intervals, *P*-values, heterogeneity statistics, and study and participant counts are provided in Supplementary Table S1.

Patients with hypothyroidism reported significantly worse outcomes than healthy controls, particularly in domains such as tiredness (SMD = 0.829, *P* < 0.001), emotional susceptibility (SMD = 0.669, *P* < 0.001), depressive symptoms (SMD = 0.514, *P* < 0.001), anxiety (SMD = 0.520, *P* < 0.001), and cognitive complaints (SMD = 0.552, *P* < 0.001). Eye symptoms and hypothyroid-related complaints were also elevated compared with controls. In contrast, domains related to goiter symptoms and impaired social or daily life did not show statistically significant differences.

Compared with hypothyroidism, patients with non-toxic goiter had significantly lower ThyPRO scores in tiredness, emotional susceptibility, impaired social life, impaired daily life, and impaired sex life. In comparisons with healthy controls, non-toxic goiter was associated with higher scores in several symptom domains, including goiter symptoms. Data were unavailable for some functional and cosmetic domains (Supplementary Table S1).

In comparisons between toxic nodular goiter and hypothyroidism, emotional susceptibility differed significantly (SMD = −0.306, *P* = 0.003), whereas the other assessed domains did not show statistically significant differences (Supplementary Table S1).

When comparing Graves’ disease to hypothyroidism, patients with Graves’ disease reported significantly more hyperthyroid symptoms (SMD = 0.438, *P* = 0.014) and fewer hypothyroid symptoms (SMD = −0.270, *P* < 0.001). Interestingly, cognitive complaints were significantly lower in Graves’ disease compared with hypothyroidism, while most other domains, including overall QoL, did not differ significantly. Similar patterns were seen in Graves’ orbitopathy compared with hypothyroidism, where hyperthyroid symptoms were higher (SMD = 0.288, *P* = 0.011), but only impaired daily life and impaired sex life showed significant increases; most other domains, including emotional and psychological symptoms, were comparable.

In comparisons between toxic nodular goiter and non-toxic goiter, goiter symptom scores were lower in toxic nodular goiter (SMD = −0.175, *P* = 0.039), whereas hyperthyroid symptom scores (SMD = 0.288, *P* = 0.010) and cosmetic complaint scores (SMD = 0.205, *P* = 0.016) were higher. Other assessed domains did not differ significantly. In comparisons between Graves’ disease and non-toxic goiter, differences were observed in several disease-specific, emotional, and functional domains (Supplementary Table S1).

Finally, comparisons within subgroups of Graves’ disease revealed that patients with Graves’ orbitopathy experienced significantly more eye-related complaints than those with Graves without orbitopathy (SMD = 0.945, *P* < 0.001), along with greater impairments in daily and sex life, and higher cosmetic concerns. However, no significant differences were found in mood or cognitive symptoms between these subgroups.

## Discussion

In this meta-analysis, QoL in benign thyroid diseases was assessed using the ThyPRO questionnaire. The pooled pre-post-treatment analyses showed statistically significant improvements in several QoL domains within each disease group. However, the magnitude and statistical significance of the estimates varied across domains, and substantial heterogeneity was present in several analyses. Accordingly, the findings are best interpreted as descriptive evidence of treatment-associated change within disease groups rather than as a ranking of treatment efficacy across thyroid conditions.

In Graves’ disease, statistically significant pre-post-treatment improvements were observed across multiple ThyPRO domains, including hyperthyroid symptoms (SMD = −1.09, *P* < 0.001), overall QoL (SMD = −0.91, *P* < 0.001), impaired daily life, anxiety, emotional susceptibility, and depressive symptoms, whereas tiredness did not change significantly. These findings are consistent with previous studies reporting improvement in QoL following treatment of Graves’ disease (14, 36, 37). Because the included studies used different treatment modalities and were not designed for head-to-head comparisons of treatment efficacy, these results should not be interpreted as demonstrating superiority of one treatment strategy over another.

In non-toxic goiter, significant pre-post-treatment improvements were observed in all assessed ThyPRO domains, including goiter symptoms (SMD = −1.48, *P* < 0.001), overall QoL, cosmetic complaints, anxiety, tiredness, and impaired daily life. Previous studies have similarly reported improved QoL after thyroidectomy, radioactive iodine treatment, and ethanol sclerotherapy (15, 25). One possible explanation for changes in goiter-related domains is that reduction in goiter volume may alleviate local symptoms such as neck pressure and swallowing difficulties. This mechanism was not directly evaluated in the present meta-analysis and should, therefore, be regarded as a clinical hypothesis rather than an inference from the pooled data.

In toxic nodular goiter, significant pre-post-treatment improvements were observed in hyperthyroid symptoms (SMD = −1.05, *P* = 0.012), goiter symptoms (SMD = −1.59, *P* = 0.013), overall QoL, tiredness, anxiety, depressive symptoms, and several other domains, whereas cognitive complaints, emotional susceptibility, and impaired social life did not change significantly. Similar patterns have been reported by Cramon *et al.* (16). Reductions in goiter volume and control of thyroid hormone excess may plausibly contribute to improvement in disease-specific symptoms; however, these mechanisms were not directly tested in the included pooled analyses. Interpretation should also account for the substantial heterogeneity in several toxic nodular goiter estimates.

In hypothyroidism, significant pre-post-treatment improvements were observed in selected domains, including tiredness (SMD = −0.54, *P* < 0.001), overall QoL (SMD = −0.73, *P* < 0.001), impaired daily life, emotional susceptibility, anxiety, depressive symptoms, and cognitive complaints, whereas goiter symptoms, hyperthyroid symptoms, and impaired social life did not change significantly. Winther *et al.* similarly reported improvement in several QoL domains during levothyroxine treatment (33). Persistent symptoms despite biochemical euthyroidism may have multiple explanations, including comorbidity, body mass index, autoimmune activity, and individual differences in thyroid hormone metabolism (38, 39, 40, 41, 42, 43, 44, 45). These potential mechanisms were not directly evaluated in the present meta-analysis and should be interpreted cautiously. Current meta-analytic evidence does not demonstrate superior QoL with T4/T3 combination therapy compared with T4 monotherapy (46, 47).

Persistent QoL impairment after treatment may also reflect factors not specific to thyroid function. ThyPRO is patient-reported, and health perception may be influenced by disease awareness and coexisting conditions (48, 49, 50). In treated hyperthyroidism, post-treatment weight gain and iatrogenic hypothyroidism may also contribute to persistent symptoms and impaired QoL (51, 52, 53, 54, 55). Psychosocial factors, including socioeconomic status, interpersonal relationships, and personality traits, may further influence patient-reported outcomes. These considerations support a broad clinical assessment of persistent symptoms rather than attributing all residual QoL impairment to thyroid dysfunction alone.

Analyses comparing ThyPRO domains between patient groups identified statistically significant differences in selected domains (Supplementary Table S1). For example, hyperthyroid symptom scores differed between Graves’ disease and hypothyroidism, and goiter-related symptom scores differed between toxic and non-toxic goiter. Patients with Graves’ orbitopathy reported more eye-related complaints than patients with Graves’ disease without orbitopathy. These comparisons describe differences in symptom profiles and should not be interpreted as evidence that treatment is more or less effective in one thyroid condition than another, because treatment effects were derived from separate and heterogeneous study pools. Potential pathophysiological explanations for differences in cognitive, emotional, sexual, and eye-related domains have been proposed (56, 57, 58, 59, 60, 61, 62), but these mechanisms were not directly evaluated in this meta-analysis and remain hypothesis-generating.

### Strengths

A major strength of this study is that it is among the first meta-analyses to synthesize evidence from multiple QoL studies in benign thyroid diseases using the ThyPRO tool. Unlike studies focused on a single disease, our analysis summarizes data across several thyroid conditions while retaining disease-specific pooled estimates. This approach provides clinicians with an overview of the QoL domains in which statistically significant treatment-associated changes have been reported and highlights domains in which changes were less consistent or not statistically significant.

### Limitations

This study has several limitations that should be considered when interpreting the findings. First, substantial heterogeneity was observed across many pooled analyses, likely reflecting differences in study design, patient populations, baseline disease severity, treatment modalities, treatment indications, and follow-up duration. Although random-effects models account for between-study variability in the estimation of pooled effects, they do not eliminate the underlying clinical and methodological heterogeneity. Consequently, heterogeneity reduces the precision and robustness of some pooled estimates and limits their generalizability to specific patient groups. Second, many included studies were observational or non-randomized, which limits causal inference and leaves the findings susceptible to confounding, selection bias, and differences in treatment allocation. The observed changes should, therefore, be interpreted as descriptive associations rather than evidence of causal treatment effects or comparative treatment efficacy. Third, reporting of ThyPRO outcomes varied across studies; not all studies reported all domains, and some summary statistics required transformation or estimation, introducing additional uncertainty. The number of contributing studies was small for several analyses, which further limits precision. Fourth, although publication bias was assessed using Egger’s test, the small number of studies in several analyses limits the reliability of this test, and small-study effects cannot be excluded. Finally, the inclusion of different thyroid conditions and treatment approaches broadens the scope of the review but limits the specificity of conclusions for individual patient populations.

### Future perspectives

Given the variety of treatment methods, further prospective studies are needed to assess QoL in well-defined patient populations and in relation to specific treatments. Studies designed for direct treatment comparisons would help determine whether observed differences in QoL changes reflect treatment effects rather than differences in disease characteristics, study design, or patient selection. Further work should also focus on patients with persistent impairment in specific ThyPRO domains despite treatment and evaluate targeted interventions using repeated QoL assessment.

## Conclusion

Treatment was associated with improvements in overall QoL and several symptom domains across the analyzed benign thyroid conditions. In Graves’ disease and toxic nodular goiter, significant changes were observed in hyperthyroid-related symptoms; in non-toxic goiter, significant changes were observed in goiter symptoms and cosmetic complaints; and in hypothyroidism, significant changes were observed in tiredness, emotional domains, and overall QoL. Because the underlying evidence derives from heterogeneous study pools and does not provide head-to-head comparisons of treatment efficacy across thyroid conditions, these findings should be interpreted descriptively rather than as a ranking of treatment effects. Disease-specific QoL assessment may help identify symptom domains that remain clinically relevant during follow-up.

## Supplementary materials





## Declaration of interest

The authors declare that there is no conflict of interest that could be perceived as prejudicing the impartiality of the work reported.

## Funding

The authors declare that no funds, grants, or other support were received during the preparation of this manuscript.

## Author contribution statement

NSG, ADR, BB, and AK conceptualized this study. NSG, ADR, BB, and KN designed the methodology. JM, KN, and AK conducted formal analysis and investigation. PK, JM, OA, and ZR wrote the original draft of this article. NSG, KN, PK, and AK wrote, reviewed, and edited this work. NSG, ADR, BB, and MR dealt with resources. NSG, ADR, BB, MR, and KN supervised this study. All authors read and approved the final manuscript.

## Data availability

The database can be provided upon request.
