# Supplementary material for: Health-related quality of life in benign thyroid diseases assessed by ThyPRO
Source: Endocr Connect. 2026 Oct 27;15(10):e260097. doi: 10.1530/EC-26-0097 (PMC13726393; doi:10.1530/EC-26-0097)
Supplement: Supplementary file 1 [file EC-26-0097_supplementary_figure_1.pdf]

Risk of bias

Study	D1	D2	D3	D4	D5	D6	D7	Overall
Azaria et al., 2022 [11]	+	+	-	+	+	+	+	+
Bukvic et al., 2014 [12]	+	-	X	+	+	+	+	-
Bukvic et al., 2015[13]	+	+	-	+	+	+	+	+
Bukvic et al., 2015 [14]	+	-	-	+	+	-	+	-
Cramon et al., 2015 [15]	+	+	X	-	+	+	+	-
Cramon et al., 2016 [16]	+	+	+	-	+	+	+	+
Johri et al., 2020 [17]	+	+	-	+	+	-	+	-
Martino et al., 2021 [18]	+	+	X	-	-	+	+	-
Michaelsson et al., 2018 [19]	+	+	-	+	+	+	+	-
Mishra et al., 2013 [20]	+	+	-	+	-	+	+	+
Molewijk et al., 2024 [21]	+	-	+	-	-	+	+	-
Oddo et al., 2018 [22]	+	-	-	-	-	+	+	-
Recker et al., 2019 [23]	+	-	-	+	+	+	+	+
Rigó et al., 2023 [24]	+	-	+	-	+	-	+	-
Sorensen et al., 2017 [25]	+	+	+	+	+	-	+	+
Sorensen et al., 2017 [26]	+	-	-	+	+	+	+	+
Sorensen et al., 2022 [27]	+	+	+	+	+	+	+	+
Taïeb et al., 2016 [28]	+	-	-	+	+	+	-	-
Törring et al., 2019 [29]	+	-	X	+	+	+	-	-
Tuzil et al., 2023 [30]	+	-	-	-	+	+	-	-
Watt et al., 2014 [31]	+	+	-	-	+	+	+	+
Watt et al., 2014 [32]	+	+	+	+	+	+	+	+
Winther et al., 2016 [33]	+	+	X	+	+	+	+	-
Zahan et al., 2018 [34]	+	-	X	+	+	+	+	-
Zivaljevic et al., 2015 [35]	+	+	-	+	+	+	+	+

D1: Study question clearly stated
D2: Eligibility criteria prespecified
D3: Sample size justification
D4: Intervention clearly described
D5: Outcome measures valid and reliable
D6: Outcomes measured consistently
D7: Statistical methods appropriate

Judgement
X High
- Unclear
+ Low
