# Supplementary material for: Health-related quality of life in benign thyroid diseases assessed by ThyPRO
Source: Endocr Connect. 2026 Oct 27;15(10):e260097. doi: 10.1530/EC-26-0097 (PMC13726393; doi:10.1530/EC-26-0097)
Supplement: Supplementary file 2 [file EC-26-0097_supplementary_table_1.pdf]

Supplementary Table 1 Comparative pooled standardised mean differences for ThyPRO domains across thyroid conditions**.**

ThyPRO domain	SMD	SE	95% CI	p-value	I^2	k	N1	N2	ref.	Egger's test
Hypothyroidism (interv) vs controls (ctrl)										
Goiter symptoms	0.351	0.320	-0.277; 0.978	0.273	89.88	3	160	829	[19, 31, 34]	0.476
Hyperthyroid symptoms	0.138	0.260	-0.373; 0.649	0.597	84.49	3	160	829	[19, 31, 34]	0.158
Hypothyroid symptoms	0.488	0.171	0.153; 0.824	0.004*	79.06	4	238	1583	[19, 20, 31, 34]	0.136
Eye symptoms	0.415	0.109	0.201; 0.628	<0.001*	22.39	3	160	829	[19, 31, 34]	0.316
Tiredness	0.829	0.165	0.506; 1.152	<0.001*	87.84	5	1905	1860	[19, 20, 22, 31, 34]	0.091
Cognitive complaints	0.552	0.157	0.244; 0.860	<0.001*	86.80	5	1905	1860	[19, 20, 22, 31, 34]	0.004^
Anxiety	0.520	0.091	0.341; 0.698	<0.001*	60.39	5	1905	1860	[19, 20, 22, 31, 34]	0.040^

Depressive symptoms	0.514	0.085	0.348; 0.680	<0.001*	54.96	5	1905	1860	[19, 20, 22, 31, 34]	0.037^
Emotional susceptibility	0.669	0.115	0.444; 0.895	<0.001*	74.83	5	1905	1860	[19, 20, 22, 31, 34]	0.169
Impaired social life	0.435	0.350	-0.250; 1.121	0.213	93.71	5	1905	1860	[19, 20, 22, 31, 34]	0.261
Impaired daily life	0.221	0.396	-0.555; 0.998	0.576	95.13	3	1749	467	[19, 22, 31]	0.214
Impaired sex life	n/a									
Cosmetic complaints	0.291	0.136	0.024; 0.558	0.033*	0.00	2	82	190	[19, 31]	<0.001^
Overall QOL	0.558	0.341	-0.111; 1.228	0.102	93.37	3	1749	467	[19, 22, 31]	0.441
Non-toxic goiter (interv) vs controls (ctrl)										
Goiter symptoms	3.083	0.379	2.341; 3.825	<0.001*	96.04	3	248	7080	[16, 26, 28]	0.377
Hyperthyroid symptoms	0.466	0.105	0.259; 0.672	<0.001*	59.01	3	248	7080	[16, 26, 28]	0.277
Hypothyroid symptoms	0.281	0.123	0.040; 0.522	0.022*	69.9	3	248	7080	[16, 26, 28]	0.159

					3					
Eye symptoms	n/a									
Tiredness	0.181	0.417	-0.637; 0.999	0.665	97.38	3	248	7080	[16, 26, 28]	0.825
Cognitive complaints	0.380	0.087	0.209; 0.551	<0.001*	40.76	3	248	7080	[16, 26, 28]	0.510
Anxiety	0.635	0.207	0.228; 1.041	0.002*	89.23	3	248	7080	[16, 26, 28]	0.917
Depressive symptoms	0.391	0.107	0.182; 0.600	<0.001*	59.95	3	248	7080	[16, 26, 28]	0.870
Emotional susceptibility	0.463	0.113	0.242; 0.684	<0.001*	64.17	3	248	7080	[16, 26, 28]	0.782
Impaired social life	n/a									
Impaired daily life	n/a									
Impaired sex life	n/a									
Cosmetic complaints	n/a									
Overall QOL	n/a									
Non-toxic goiter (interv) vs hypothyroidism (ctrl)										
Goiter symptoms	0.321	0.280	-0.230; 0.871	0.253	90.71	5	528	353	[14, 32, 33, 35, 36]	0.427
Hyperthyroid	0.06	0.07	-0.087;	0.399	7.	5	52	35	[14, 32, 33, 35,	0.341

symptoms	6	8	0.219		84		8	3	36]	
Hypothyroid symptoms	-0.258	0.143	-0.538; 0.022	0.071	64.62	5	528	353	[14, 32, 33, 35, 36]	0.469
Eye symptoms	-0.045	0.072	-0.186; 0.095	0.528	0.00	5	528	353	[14, 32, 33, 35, 36]	0.148
Tiredness	-0.203	0.084	-0.368; -0.039	0.016*	14.61	5	528	353	[14, 32, 33, 35, 36]	0.265
Cognitive complaints	-0.135	0.134	-0.397; 0.128	0.314	60.07	5	528	353	[14, 32, 33, 35, 36]	0.001^
Anxiety	0.139	0.131	-0.118; 0.396	0.288	58.33	5	528	353	[14, 32, 33, 35, 36]	0.932
Depressive symptoms	-0.125	0.072	-0.265; 0.015	0.081	0.00	5	528	353	[14, 32, 33, 35, 36]	0.839
Emotional susceptibility	-0.161	0.072	-0.301; -0.020	0.025*	0.00	5	528	353	[14, 32, 33, 35, 36]	0.318
Impaired social life	-0.238	0.072	-0.379; -0.097	0.001*	0.00	5	528	353	[14, 32, 33, 35, 36]	0.253
Impaired daily life	-0.33	0.074	-0.478; -0.189	<0.001*	2.07	5	528	353	[14, 32, 33, 35, 36]	0.397

	3									
Impaired sex life	-0.257	0.072	-0.398; -0.116	<0.001*	0.00	5	528	353	[14, 32, 33, 35, 36]	0.293
Cosmetic complaints	-0.229	0.110	-0.444; -0.013	0.037*	42.54	5	528	353	[14, 32, 33, 35, 36]	0.675
Overall QOL	-0.189	0.131	-0.448; 0.070	0.151	0.00	2	178	111	[32, 36]	<0.001^
Toxic nodular goiter (interv) vs hypothyroidism (ctrl)										
Goiter symptoms	0.131	0.219	-0.299; 0.561	0.550	45.47	3	171	242	[14, 33, 35]	0.161
Hyperthyroid symptoms	0.201	0.155	-0.103; 0.505	0.195	19.31	3	171	242	[14, 33, 35]	0.690
Hypothyroid symptoms	-0.233	0.236	-0.697; 0.231	0.324	51.31	3	171	242	[14, 33, 35]	0.515
Eye symptoms	-0.113	0.103	-0.315; 0.090	0.274	0.00	3	171	242	[14, 33, 35]	0.377
Tiredness	0.047	0.230	-0.406; 0.499	0.839	49.43	3	171	242	[14, 33, 35]	0.278
Cognitive complaints	0.05	0.31	-0.555;	0.863	69	3	17	24	[14, 33, 35]	0.105

	3	0	0.662		.63		1	2		
Anxiety	-0.036	0.103	-0.238; 0.166	0.727	0.00	3	171	242	[14, 33, 35]	0.177
Depressive symptoms	-0.174	0.103	-0.376; 0.028	0.092	0.00	3	171	242	[14, 33, 35]	0.416
Emotional susceptibility	-0.306	0.103	-0.510; -0.103	0.003*	0.00	3	171	242	[14, 33, 35]	0.088
Impaired social life	-0.091	0.103	-0.293; 0.112	0.379	0.00	3	171	242	[14, 33, 35]	0.733
Impaired daily life	-0.086	0.103	-0.288; 0.117	0.406	0.00	3	171	242	[14, 33, 35]	0.997
Impaired sex life	-0.142	0.103	-0.344; 0.061	0.169	0.00	3	171	242	[14, 33, 35]	0.739
Cosmetic complaints	-0.135	0.142	-0.414; 0.144	0.341	14.24	3	171	242	[14, 33, 35]	0.964
Overall QOL	n/a									
Graves' disease (interv) vs hypothyroidism (ctrl)										
Goiter symptoms	0.027	0.151	-0.270; 0.324	0.857	59.5	5	280	566	[14, 25, 32, 33, 35]	0.590

					6					
Hyperthyroid symptoms	0.438	0.178	0.090; 0.787	0.014*	68.99	5	280	566	[14, 25, 32, 33, 35]	0.623
Hypothyroid symptoms	-0.270	0.077	-0.421; -0.119	<0.001*	0.00	5	280	566	[14, 25, 32, 33, 35]	0.326
Eye symptoms	0.167	0.151	-0.129; 0.464	0.268	59.42	5	280	566	[14, 25, 32, 33, 35]	0.656
Tiredness	-0.035	0.087	-0.205; 0.135	0.687	11.08	5	280	566	[14, 25, 32, 33, 35]	0.120
Cognitive complaints	-0.212	0.077	-0.362; -0.061	0.006*	0.00	5	280	566	[14, 25, 32, 33, 35]	0.585
Anxiety	0.230	0.121	-0.009; 0.468	0.059	42.11	5	280	566	[14, 25, 32, 33, 35]	0.606
Depressive symptoms	0.083	0.077	-0.068; 0.233	0.280	0.00	5	280	566	[14, 25, 32, 33, 35]	0.245
Emotional susceptibility	0.106	0.077	-0.045; 0.256	0.168	0.00	5	280	566	[14, 25, 32, 33, 35]	0.917
Impaired social life	0.024	0.077	-0.126; 0.174	0.753	0.00	5	280	566	[14, 25, 32, 33, 35]	0.243
Impaired daily life	0.006	0.077	-0.144; 0.156	0.937	0.00	5	280	566	[14, 25, 32, 33, 35]	0.683

Impaired sex life	0.044	0.088	-0.130; 0.217	0.622	0.00	4	229	326	[14, 32, 33, 35]	0.611
Cosmetic complaints	0.038	0.144	-0.245; 0.320	0.794	56.24	5	280	566	[14, 25, 32, 33, 35]	0.788
Overall QOL	0.054	0.112	-0.167; 0.274	0.632	0.00	2	117	324	[25, 32]	<0.001^
Graves' orbitopathy (interv) vs hypothyroidism (ctrl)										
Goiter symptoms	-0.047	0.112	-0.267; 0.174	0.677	0.00	3	127	242	[14, 33, 35]	0.843
Hyperthyroid symptoms	0.288	0.112	0.067; 0.509	0.011*	0.00	3	127	242	[14, 33, 35]	0.467
Hypothyroid symptoms	0.016	0.112	-0.204; 0.236	0.885	0.00	3	127	242	[14, 33, 35]	0.363
Eye symptoms	0.553	0.476	-0.384; 1.489	0.246	89.32	3	127	242	[14, 33, 35]	0.635
Tiredness	0.182	0.112	-0.038; 0.402	0.105	0.00	3	127	242	[14, 33, 35]	0.741
Cognitive complaints	0.020	0.112	-0.200; 0.240	0.856	0.00	3	127	242	[14, 33, 35]	0.573
Anxiety	0.169	0.112	-0.052; 0.389	0.134	0.00	3	127	242	[14, 33, 35]	0.567
Depressive symptoms	0.104	0.112	-0.116; 0.325	0.352	0.00	3	127	242	[14, 33, 35]	0.570
Emotional	0.07	0.11	-0.146;	0.509	0.	3	12	24	[14, 33, 35]	0.013^

susceptibility	4	2	0.294		00		7	2		
Impaired social life	0.180	0.112	-0.040; 0.401	0.108	0.00	3	127	242	[14, 33, 35]	0.332
Impaired daily life	0.310	0.113	0.088; 0.531	0.006*	0.00	3	127	242	[14, 33, 35]	0.186
Impaired sex life	0.230	0.112	0.009; 0.451	0.041*	0.00	3	127	242	[14, 33, 35]	0.286
Cosmetic complaints	0.258	0.397	-0.524; 1.039	0.517	85.12	3	127	242	[14, 33, 35]	0.178
Overall QOL	n/a									
Toxic nodular goiter (interv) vs non-toxic goiter (ctrl)										
Goiter symptoms	-0.175	0.085	-0.342; -0.009	0.039*	0.00	4	210	427	[13, 14, 33, 35]	0.066
Hyperthyroid symptoms	0.288	0.111	0.069; 0.506	0.010*	23.39	4	210	427	[13, 14, 33, 35]	0.513
Hypothyroid symptoms	-0.009	0.084	-0.175; 0.156	0.912	0.00	4	210	427	[13, 14, 33, 35]	0.721
Eye symptoms	0.000	0.084	-0.165; 0.166	0.997	0.00	4	210	427	[13, 14, 33, 35]	0.860
Tiredness	0.135	0.085	-0.031; 0.301	0.111	0.00	4	210	427	[13, 14, 33, 35]	0.062
Cognitive complaints	0.089	0.084	-0.077; 0.255	0.293	0.00	4	210	427	[13, 14, 33, 35]	0.446

Anxiety	-0.044	0.084	-0.210; 0.122	0.603	0.00	4	210	427	[13, 14, 33, 35]	0.737
Depressive symptoms	-0.094	0.084	-0.260; 0.072	0.265	0.00	4	210	427	[13, 14, 33, 35]	0.676
Emotional susceptibility	-0.293	0.184	-0.653; 0.068	0.112	66.83	4	210	427	[13, 14, 33, 35]	0.676
Impaired social life	0.262	0.148	-0.028; 0.552	0.077	50.33	4	210	427	[13, 14, 33, 35]	0.938
Impaired daily life	0.133	0.122	-0.107; 0.373	0.276	32.89	4	210	427	[13, 14, 33, 35]	0.530
Impaired sex life	0.111	0.085	-0.054; 0.277	0.188	0.00	4	210	427	[13, 14, 33, 35]	0.398
Cosmetic complaints	0.205	0.085	0.039; 0.371	0.016*	0.00	4	210	427	[13, 14, 33, 35]	0.009^
Overall QOL	n/a									
Graves' disease (interv) vs non-toxic goiter (ctrl)										
Goiter symptoms	-0.660	0.088	-0.833; -0.487	<0.001*	0.00	4	229	412	[14, 32, 33, 35]	0.946
Hyperthyroid symptoms	0.481	0.146	0.195; 0.767	0.001*	33.54	4	229	412	[14, 32, 33, 35]	0.426

Hypothyroid symptoms	0.128	0.086	-0.041; 0.297	0.137	0.00	4	229	412	[14, 32, 33, 35]	0.980
Eye symptoms	0.131	0.086	-0.038; 0.300	0.128	0.00	4	229	412	[14, 32, 33, 35]	0.272
Tiredness	0.275	0.128	0.024; 0.525	0.032*	24.19	4	229	412	[14, 32, 33, 35]	0.395
Cognitive complaints	0.167	0.086	-0.002; 0.336	0.053	0.00	4	229	412	[14, 32, 33, 35]	0.346
Anxiety	0.126	0.086	-0.043; 0.295	0.144	0.00	4	229	412	[14, 32, 33, 35]	0.264
Depressive symptoms	0.191	0.086	0.022; 0.360	0.027*	0.00	4	229	412	[14, 32, 33, 35]	0.186
Emotional susceptibility	0.302	0.086	0.132; 0.471	0.001*	0.00	4	229	412	[14, 32, 33, 35]	0.573
Impaired social life	0.253	0.086	0.083; 0.422	0.004*	0.00	4	229	412	[14, 32, 33, 35]	0.251
Impaired daily life	0.399	0.087	0.229; 0.570	<0.001*	0.00	4	229	412	[14, 32, 33, 35]	0.804
Impaired sex life	0.346	0.087	0.176; 0.516	<0.001*	0.00	4	229	412	[14, 32, 33, 35]	0.136
Cosmetic complaints	0.136	0.121	-0.102; 0.374	0.262	20.87	4	229	412	[14, 32, 33, 35]	0.781
Overall QOL	n/a									
Graves' orbitopathy (interv) vs non-toxic goiter (ctrl)										
Goiter symptoms	-	0.29	-0.766;	0.529	81	3	12	35	[14, 33, 35]	0.205

	0.186	5	0.394		.13		7	0		
Hyperthyroid symptoms	0.310	0.104	0.107; 0.514	0.003*	0.00	3	127	350	[14, 33, 35]	0.479
Hypothyroid symptoms	0.334	0.115	0.108; 0.560	0.004*	8.65	3	127	350	[14, 33, 35]	0.579
Eye symptoms	0.653	0.382	-0.097; 1.403	0.088	88.21	3	127	350	[14, 33, 35]	0.490
Tiredness	0.367	0.104	0.163; 0.571	<0.001*	0.00	3	127	350	[14, 33, 35]	0.287
Cognitive complaints	0.108	0.203	-0.291; 0.507	0.596	61.18	3	127	350	[14, 33, 35]	0.153
Anxiety	0.149	0.103	-0.054; 0.352	0.150	0.00	3	127	350	[14, 33, 35]	0.534
Depressive symptoms	0.191	0.103	-0.012; 0.394	0.065	0.00	3	127	350	[14, 33, 35]	0.242
Emotional susceptibility	0.232	0.103	0.029; 0.435	0.025*	0.00	3	127	350	[14, 33, 35]	0.751
Impaired social life	0.341	0.170	0.007; 0.675	0.046*	46.39	3	127	350	[14, 33, 35]	0.163
Impaired daily life	0.485	0.210	0.072; 0.897	0.021*	63.02	3	127	350	[14, 33, 35]	0.133
Impaired sex life	0.39	0.22	-0.041;	0.075	67	3	12	35	[14, 33, 35]	0.235

	8	3	0.837		.24		7	0		
Cosmetic complaints	0.735	0.321	0.104; 1.367	0.023*	83.56	3	127	350	[14, 33, 35]	0.218
Overall QOL	n/a									
Toxic nodular goiter (interv) vs Graves' disease (ctrl)										
Goiter symptoms	0.301	0.092	0.120; 0.483	0.001*	0.00	4	239	251	[15, 17, 33, 35]	0.640
Hyperthyroid symptoms	-0.325	0.333	-0.979; 0.330	0.330	84.18	4	239	251	[15, 17, 33, 35]	0.717
Hypothyroid symptoms	-0.256	0.131	-0.514; 0.002	0.052	26.20	4	239	251	[15, 17, 33, 35]	0.920
Eye symptoms	-0.150	0.092	-0.331; 0.031	0.104	0.00	4	239	251	[15, 17, 33, 35]	0.297
Tiredness	-0.295	0.231	-0.748; 0.159	0.203	68.09	4	239	251	[15, 17, 33, 35]	0.852
Cognitive complaints	-0.292	0.140	-0.567; -0.017	0.038*	31.28	4	239	251	[15, 17, 33, 35]	0.736
Anxiety	-0.166	0.092	-0.346; 0.015	0.073	0.00	4	239	251	[15, 17, 33, 35]	0.663

Depressive symptoms	-0.310	0.092	-0.491; -0.128	0.001*	0.00	4	239	251	[15, 17, 33, 35]	0.504
Emotional susceptibility	-0.506	0.093	-0.689; -0.322	<0.001*	0.00	4	239	251	[15, 17, 33, 35]	0.898
Impaired social life	-0.288	0.184	-0.650; 0.074	0.118	53.01	4	239	251	[15, 17, 33, 35]	0.653
Impaired daily life	-0.336	0.276	-0.877; 0.206	0.223	77.25	4	239	251	[15, 17, 33, 35]	0.849
Impaired sex life	-0.113	0.092	-0.294; 0.067	0.218	0.00	4	239	251	[15, 17, 33, 35]	0.372
Cosmetic complaints	-0.144	0.126	-0.392; 0.104	0.254	23.17	4	239	251	[15, 17, 33, 35]	0.747
Overall QOL	n/a									
Graves' orbitopathy (interv) vs Graves' disease (ctrl)										
Goiter symptoms	0.014	0.218	-0.415; 0.444	0.948	15.68	3	127	163	[14, 33, 35]	0.253
Hyperthyroid symptoms	0.073	0.311	-0.540; 0.685	0.816	36.16	3	127	163	[14, 33, 35]	0.445
Hypothyroid	0.29	0.12	0.046;	0.021*	0.	3	12	16	[14, 33, 35]	0.188

symptoms	5	7	0.545		00		7	3		
Eye symptoms	0.945	0.169	0.613; 1.277	<0.001*	5.43	3	127	163	[14, 33, 35]	0.582
Tiredness	0.178	0.218	-0.251; 0.608	0.415	15.80	3	127	163	[14, 33, 35]	0.325
Cognitive complaints	0.117	0.126	-0.131; 0.366	0.353	0.00	3	127	163	[14, 33, 35]	0.583
Anxiety	0.017	0.126	-0.231; 0.265	0.892	0.00	3	127	163	[14, 33, 35]	0.148
Depressive symptoms	0.059	0.126	-0.190; 0.307	0.643	0.00	3	127	163	[14, 33, 35]	0.554
Emotional susceptibility	-0.046	0.126	-0.294; 0.202	0.716	0.00	3	127	163	[14, 33, 35]	0.779
Impaired social life	0.201	0.127	-0.048; 0.450	0.113	0.00	3	127	163	[14, 33, 35]	0.009^
Impaired daily life	0.353	0.127	0.102; 0.603	0.006*	0.00	3	127	163	[14, 33, 35]	0.071^
Impaired sex life	0.307	0.127	0.058; 0.557	0.016*	0.00	3	127	163	[14, 33, 35]	0.974
Cosmetic complaints	0.837	0.307	0.232; 1.442	0.007*	34.96	3	127	163	[14, 33, 35]	0.568
Overall QOL	n/a									

Legend: SMD, standardised mean difference (the value for the group labeled as the intervention group was reduced by the value for the group labeled as the control group); SE, standard error; CI, confidence interval; k, number of contributing studies, N1, number of participants in intervention group; N2, number of participants in control group; QoL, quality of life; *, significant pooled standardised mean differences for $p < 0.05$, ^ significant publication bias for $p < 0.05$.
